# Supplementary material for: Remote Monitoring of Hypertension Diseases in Pregnancy: A Pilot Study
Source: JMIR Mhealth Uhealth. 2017 Mar 9;5(3):e25. doi: 10.2196/mhealth.6552 (PMC5364324; doi:10.2196/mhealth.6552)
Supplement: Multimedia Appendix 1 [file mhealth_v5i3e25_app1.pdf]

**Supplementary file 1:** Multivariable model for the prediction of prenatal follow-up using maternal demographics and characteristics				
Variable	*Beta*	*95.0% CI for B*		*P-value*
		Lower Bound	*Upper Bound*	
**Outcome 1:** total number of prenatal visits				
RM vs. no RM	-0.56	-1.74	9.14	0.54
Maternal age, 1 year increase	0.13	-0.03	0.23	0.12
Pre-pregnancy weight, 1 kg increase	-1.68	-0.63	0.02	0.07
Height, 1 cm increase	0.48	-0.07	0.56	0.12
BMI, 1 kg/m² increase	1.65	-0.26	1.81	0.06
Primigravida vs. multigravida	0.04	-1.01	1.55	0.67
Smoking vs. no smoking	0.01	-2.42	2.71	0.91
GA first visit, 1 week increase	-0.45	-0.36	-0.16	**< 0.001**
**Outcome 2:** total number of CTG's				
RM vs. no RM	-0.08	-1.12	0.53	0.48
Maternal age, 1 year increase	0.09	-0.05	0.12	0.39
Pre-pregnancy weight, 1 kg increase	1.94	-0.06	0.40	0.15
Height, 1 cm increase	-0.72	-0.42	0.02	0.08
BMI, 1 kg/m² increase	-1.83	-1.13	0.17	0.15
Primigravida vs. multigravida	-0.06	-1.00	0.57	0.59
Smoking vs. no smoking	-0.19	-3.20	0.18	0.08
GA first visit, 1 week increase	0.03	-0.05	0.06	0.80
**Outcome 3:** total number of echo's				
RM vs. no RM	0.07	-0.56	1.19	0.48
Maternal age, 1 year increase	0.05	-0.06	0.11	0.60
Pre-pregnancy weight, 1 kg increase	-0.20	-0.23	0.19	0.84
Height, 1 cm increase	0.04	-0.19	0.21	0.92
BMI, 1 kg/m² increase	0.29	-0.50	0.68	0.76
Primigravida vs. multigravida	0.10	-0.41	1.26	0.32
Smoking vs. no smoking	0.11	-0.73	2.56	0.27
GA first visit, 1 week increase	-0.28	-0.18	-0.04	**0.004**
**Outcome 4:** admission to prenatal ward				
RM vs. no RM	0.46	0.18	1.45	0.09
Maternal age, 1 year increase	0.92	0.84	1.01	0.07
Pre-pregnancy weight, 1 kg increase	1.01	0.78	1.29	0.97
Height, 1 cm increase	0.97	0.76	1.24	0.80
BMI, 1 kg/m² increase	0.97	0.48	1.97	0.94
Primigravida vs. multigravida	2.18	0.90	5.27	0.08
Smoking vs. no smoking	0.29	0.05	1.60	0.16
GA first visit, 1 week increase	1.00	0.94	1.07	0.99
**Outcome 5:** days hospitalized at the prenatal ward				
RM vs. no RM	0.10	-1.62	4.81	0.32
Maternal age, 1 year increase	-0.04	-0.40	0.27	0.70
Pre-pregnancy weight, 1 kg increase	0.77	-0.47	1.02	0.46
Height, 1 cm increase	-0.35	-1.07	0.36	0.37
BMI, 1 kg/m² increase	-0.56	-2.64	1.47	0.51
Primigravida vs. multigravida	0.32	1.53	7.92	**0.004**
Smoking vs. no smoking	0.02	-6.44	7.95	0.84
GA first visit, 1 week increase	0.26	0.07	0.55	**0.01**
**Outcome 6:** prenatal admission until delivery				
RM vs. no RM	0.38	0.12	1.22	0.11
Maternal age, 1 year increase	1.09	0.96	1.22	0.17
Pre-pregnancy weight, 1 kg increase	0.95	0.72	1.25	0.71
Height, 1 cm increase	1.08	0.82	1.25	0.59
BMI, 1 kg/m² increase	1.16	0.53	1.41	0.71
Primigravida vs. multigravida	1.85	0.58	2.52	0.30

Smoking vs. no smoking	0.90	0.05	5.94	0.94
GA first visit, 1 week increase	0.97	0.89	15.18	0.52
**Outcome 7:** gestational hypertension as gestational outcome				
RM vs. no RM	6.62	2.40	18.27	**< 0.001**
Maternal age, 1 year increase	0.93	0.85	1.02	0.13
Pre-pregnancy weight, 1 kg increase	1.05	0.83	1.32	0.69
Height, 1 cm increase	1.01	0.81	1.26	0.94
BMI, 1 kg/m² increase	0.91	0.48	1.75	0.78
Primigravida vs. multigravida	0.72	0.30	1.75	0.47
Smoking vs. no smoking	1.62	0.30	8.87	0.58
GA first visit, 1 week increase	0.98	0.91	1.06	0.62
**Outcome 8:** pre-eclampsia as gestational outcome				
RM vs. no RM	0.24	0.08	0.71	**0.01**
Maternal age, 1 year increase	1.08	0.99	1.19	0.09
Pre-pregnancy weight, 1 kg increase	0.98	0.78	1.23	0.83
Height, 1 cm increase	1.02	0.82	1.27	0.86
BMI, 1 kg/m² increase	1.04	0.55	1.97	0.91
Primigravida vs. multigravida	2.41	0.94	6.23	0.07
Smoking vs. no smoking	1.16	0.22	6.30	0.86
GA first visit, 1 week increase	1.03	0.96	1.11	0.41

CI = Confidence interval, RM = remote monitoring, CVD = Cardiovascular disorders, dis. = disorder, GA = gestational age
