# Supplementary material for: Remote Monitoring of Hypertension Diseases in Pregnancy: A Pilot Study
Source: JMIR Mhealth Uhealth. 2017 Mar 9;5(3):e25. doi: 10.2196/mhealth.6552 (PMC5364324; doi:10.2196/mhealth.6552)
Supplement: Multimedia Appendix 2 [file mhealth_v5i3e25_app2.pdf]

Supplementary file 2: Multivariable model for the prediction of gestational outcomes using maternal demographics and characteristics

Variable	*Beta*	*95.0% CI for B*		*P-value*
		Lower Bound	*Upper Bound*	
**Outcome 1:** gestational age				
RM vs. no RM	-0.21	-1.29	1.06	0.85
Maternal age, 1 year increase	0.01	-0.11	0.12	0.95
Pre-pregnancy weight, 1 kg increase	-0.90	-0.41	0.17	0.40
Height, 1 cm increase	0.39	-0.13	0.43	0.28
BMI, 1 kg/m² increase	0.83	-0.47	1.14	0.41
Primigravida vs. multigravida	-0.08	-1.55	0.72	0.47
Smoking vs. no smoking	0.07	-1.46	3.04	0.49
GA first visit, 1 week increase	-0.07	-0.12	0.06	0.50
**Outcome 2:** spontaneous start of the birth process				
RM vs. no RM	3.25	1.36	7.78	**<0.001**
Maternal age, 1 year increase	1.02	0.93	4.11	0.73
Pre-pregnancy weight, 1 kg increase	0.92	0.74	1.14	0.42
Height, 1 cm increase	1.09	0.88	1.35	0.42
BMI, 1 kg/m² increase	1.31	0.71	2.39	0.38
Primigravida vs. multigravida	0.75	0.33	1.70	0.49
Smoking vs. no smoking	1.57	0.31	8.03	0.59
GA first visit, 1 week increase	0.99	0.94	1.06	0.84
**Outcome 3:** induced start of the birth process				
RM vs. no RM	0.36	0.14	0.89	**0.03**
Maternal age, 1 year increase	0.99	0.78	1.20	0.79
Pre-pregnancy weight, 1 kg increase	0.97	0.83	1.26	0.83
Height, 1 cm increase	1.02	0.59	1.97	0.80
BMI, 1 kg/m² increase	1.08	0.75	4.12	0.20
Primigravida vs. multigravida	1.75	0.08	2.53	0.36
Smoking vs. no smoking	0.44	0.93	1.07	0.94
GA first visit, 1 week increase	1.00	0.93	1.07	0.93
**Outcome 4:** primary section				
RM vs. no RM	0.67	0.21	2.18	0.51
Maternal age, 1 year increase	1.00	0.89	1.12	0.99
Pre-pregnancy weight, 1 kg increase	1.26	0.92	1.72	0.15
Height, 1 cm increase	0.81	0.60	1.09	0.16
BMI, 1 kg/m² increase	0.51	0.21	1.23	0.14
Primigravida vs. multigravida	0.62	0.21	1.89	0.40
Smoking vs. no smoking	1.61	0.25	10.58	0.62
GA first visit, 1 week increase	1.02	0.94	1.10	0.69
**Outcome 5:** vaginal mode of delivery				
RM vs. no RM	1.06	0.44	2.54	0.90
Maternal age, 1 year increase	0.93	0.85	1.02	0.12
Pre-pregnancy weight, 1 kg increase	0.90	0.71	1.13	0.36
Height, 1 cm increase	1.07	0.87	1.33	0.52
BMI, 1 kg/m² increase	1.39	0.73	2.65	0.32
Primigravida vs. multigravida	0.78	0.33	1.82	0.56
Smoking vs. no smoking	1.54	0.26	9.36	0.64
GA first visit, 1 week increase	1.04	0.97	1.13	0.25
**Outcome 6:** instrumental mode of delivery				
RM vs. no RM	2.34	0.47	11.64	0.30
Maternal age, 1 year increase	1.12	0.95	1.32	0.19
Pre-pregnancy weight, 1 kg increase	0.92	0.57	1.48	0.73
Height, 1 cm increase	1.21	0.79	1.84	0.39
BMI, 1 kg/m² increase	1.13	0.28	4.60	0.86
Primigravida vs. multigravida	3.02	0.50	18.37	0.23

GA first visit, 1 week increase	0.64	0.64	1.16	0.33
**Outcome 7:** secondary cesarean section				
RM vs. no RM	0.49	0.11	2.10	0.33
Maternal age, 1 year increase	1.13	0.99	1.30	0.07
Pre-pregnancy weight, 1 kg increase	1.24	0.86	1.78	0.25
Height, 1 cm increase	0.84	0.60	1.18	0.31
BMI, 1 kg/m² increase	0.56	0.20	1.56	0.27
Primigravida vs. multigravida	2.46	0.58	10.45	0.22
GA first visit, 1 week increase	0.90	0.73	1.10	0.30
**Outcome 8:** birthweight				
RM vs. no RM	0.11	-162.71	535.33	0.29
Maternal age, 1 year increase	0.02	-31.11	37.96	0.85
Pre-pregnancy weight, 1 kg increase	-1.70	-158.02	13.67	0.10
Height, 1 cm increase	0.68	-0.77	165.474	0.05
BMI, 1 kg/m² increase	1.72	-23.10	460.064	0.08
Primigravida vs. multigravida	-0.04	-404.64	267.42	0.69
Smoking vs. no smoking	0.06	-478.94	870.62	0.57
GA first visit, 1 week increase	0.07	-16.73	35.10	0.48
**Outcome 9:** length				
RM vs. no RM	0.23	0.02	3.45	**0.05**
Maternal age, 1 year increase	0.08	-0.12	0.23	0.50
Pre-pregnancy weight, 1 kg increase	-2.07	-0.83	0.01	0.06
Height, 1 cm increase	0.68	-0.04	0.76	0.08
BMI, 1 kg/m² increase	2.12	0.06	2.42	**0.04**
Primigravida vs. multigravida	0.10	-0.97	2.47	0.39
Smoking vs. no smoking	-0.01	-4.02	3.65	0.92
GA first visit, 1 week increase	0.10	-0.08	0.21	0.37
**Outcome 9:** Apgar at 1’				
RM vs. no RM	0.08	-0.38	0.88	0.43
Maternal age, 1 year increase	0.12	-0.03	0.10	0.25
Pre-pregnancy weight, 1 kg increase	-0.10	-0.16	0.015	0.93
Height, 1 cm increase	0.01	-0.15	0.15	0.98
BMI, 1 kg/m² increase	0.12	-0.41	0.46	0.91
Primigravida vs. multigravida	0.08	-0.38	0.83	0.47
Smoking vs. no smoking	0.00	-1.23	1.20	0.66
GA first visit, 1 week increase	0.04	-0.04	0.06	0.66
**Outcome 9:** Apgar at 5’				
RM vs. no RM	0.06	-0.37	0.65	0.59
Maternal age, 1 year increase	0.21	0.00	0.10	0.03
Pre-pregnancy weight, 1 kg increase	-0.18	-0.14	0.11	0.87
Height, 1 cm increase	-0.02	-0.12	0.12	0.95
BMI, 1 kg/m² increase	0.21	-0.31	0.39	0.82
Primigravida vs. multigravida	0.18	-0.44	0.53	0.85
Smoking vs. no smoking	0.06	-0.70	1.25	0.58
GA first visit, 1 week increase	0.07	-0.02	0.05	0.47
**Outcome 10:** admission to Neonatal Intensive Care				
RM vs. no RM	0.34	0.10	1.14	0.08
Maternal age, 1 year increase	1.04	0.94	1.15	0.47
Pre-pregnancy weight, 1 kg increase	0.92	0.72	1.17	0.48
Height, 1 cm increase	1.05	0.83	1.32	0.70
BMI, 1 kg/m² increase	1.31	0.67	2.57	0.43
Primigravida vs. multigravida	2.81	0.94	8.43	0.07
Smoking vs. no smoking	1.23	0.20	7.66	0.82
GA first visit, 1 week increase	1.02	0.94	1.11	**0.68**
CI = Confidence interval, RM = remote monitoring, CVD = Cardiovascular disorders, dis. = disorder, GA = gestational age				
